# A Hybrid Finite Element—Machine Learning Backward Training Approach to Analyze the Optimal Machining Conditions

**DOI:** 10.3390/ma14216717

**Published:** 2021-11-08

**Authors:** Kriz George, Sathish Kannan, Ali Raza, Salman Pervaiz

**Affiliations:** 1 Department of Mechanical and Industrial Engineering, Rochester Institute of Technology, Dubai Campus, Dubai 341055, United Arab Emirates; ktg8957@rit.edu; 2 Department of Mechanical Engineering, School of Engineering, American University of Sharjah, Sharjah 26666, United Arab Emirates; skannan@aus.edu; 3 Department of Electrical Engineering and Computing Sciences, Rochester Institute of Technology, Dubai Campus, Dubai 341055, United Arab Emirates; axrcada@rit.edu

**Keywords:** machining, AISI 630, machine learning, Artificial Neural Network

## Abstract

As machining processes are complex in nature due to the involvement of large plastic strains occurring at higher strain rates, and simultaneous thermal softening of material, it is necessary for manufacturers to have some manner of determining whether the inputs will achieve the desired outputs within the limitations of available resources. However, finite element simulations—the most common means to analyze and understand the machining of high-performance materials under various cutting conditions and environments—require high amounts of processing power and time in order to output reliable and accurate results which can lead to delays in the initiation of manufacture. The objective of this study is to reduce the time required prior to fabrication to determine how available inputs will affect the desired outputs and machining parameters. This study proposes a hybrid predictive methodology where finite element simulation data and machine learning are combined by feeding the time series output data generated by Finite Element Modeling to an Artificial Neural Network in order to acquire reliable predictions of optimal and/or expected machining inputs (depending on the application of the proposed approach) using what we describe as a backwards training model. The trained network was then fed a test dataset from the simulations, and the results acquired show a high degree of accuracy with regards to cutting force and depth of cut, whereas the predicted/expected feed rate was wildly inaccurate. This is believed to be due to either a limited dataset or the much stronger effect that cutting speed and depth of cut have on power, cutting forces, etc., as opposed to the feed rate. It shows great promise for further research to be performed for implementation in manufacturing facilities for the generation of optimal inputs or the real-time monitoring of input conditions to ensure machining conditions do not vary beyond the norm during the machining process.

## 1. Introduction

With the advent of machine learning technologies, and the rapid growth of neural networks technology in the past decade, there has been much interest shown from all industries with regards to the implementation of these networks. This paper attempts to answer the question of the most suitable circumstance under which the machining of metals, particularly AISI 630 steel, would benefit from the implementation of machine learning. The initial premise of this research was to predict time series data on the variation of forces, power, and temperature based on inputs of cutting speed, feed rate, and depth of cut. However, as these data are currently being obtained from finite element modeling, which is a process that requires a large amount of computational power, and therefore a large amount of time, we set out to reduce the time needed for manufacturers to get the results on whether the conditions under which their machining would take place would be within their allowable tolerances.

In their paper, Serin et al. (2020) [1] describe the potential for Deep Learning using the Deep Multi-Layer Perceptron (DMLP) method in the context of tool wear monitoring through the use of input parameters such as acoustic emissions, vibrations, etc., which would be measured using sensors. The paper continues to describe the potential for deep learning applications in tool condition monitoring and the shift towards Industry 4.0. In the context of this presented research, the viability of a neural network based on a deep learning model that can predict the machining performance of AISI 630 steel seems feasible, based on the estimates and predictions in the paper. Bianchini and Scarselli (2014) [2] established a new measure for the complexity of neural networks based on quirks of topology. Using this approach, it was shown that deeper networks are more suitable for solving complex problems than shallower networks when using the same amount of resources. In the context of this presented research, this raises the question as to whether the machining process can be considered a complex problem on the level of language or image processing, which are the examples of complex problems described in the paper. If this is the case then measures must be taken so that the depth of the neural network does not result in overfitting.

In the first part of a two-part paper, Dimla Sr. and Lister (2000a) [3] discuss the monitoring of tool wear conditions and the multiple parameters that contribute to tool wear. They established a methodology for measuring the forces and vibrations involved in the machining process. They then analyzed these data and compare them to the observed tool wear, finding some correlation for the parameters measured. They also describe how the parameters traditionally measured to create predictive models for tool wear do not solely affect the tool wear condition. These parameters therefore contribute towards other measurable output variables, which is a point that significantly complicates the problem at hand. In the second part of their paper, Dimla Sr. and Lister (2000b) [4] used the methods described in the previous part to predict the state of the tool being used via a neural network. The network was used to predict whether the tool was worn, sharp, or fractured/chipped by taking the output variables of flank and nose wear obtained from the neural network and generated an additional output based on these output variables on the tool state. This provides additional support for the nested neural network proposed in this paper for the prediction of more complex indicators of machining performance based on easily set initial parameters.

Kumar et al. (2019) [5] conducted studies into the machining performance of titanium alloy when subjected to Electro-Discharge Machining. They studied the surface finish of the workpiece in terms of surface roughness and surface microhardness as some of the indicators of machining performance which they describe as being heavily affected by the temperatures involved in EDM. Samy et al. (2021) [6] conducted a study into the machining performance of a boron carbide composite when subjected to turning. They considered the effect of spindle speed and feed rate on the surface finish of the workpiece, finding that at a high spindle speed the workpiece achieves a good surface finish while the tool itself is protected from abrasive wear due to the increase in temperature. They also include the friction coefficient as one of the output parameters for their analysis of the machining performance. This introduces the next output parameter to be considered for the neural network to be developed.

Vogler et al. (2005a) [7], in the first part of their two-part paper, discussed the parameters that affect the surface finish of the workpiece, which can be considered an indicator of machining performance. They described the surface roughness (at least of single-phase materials) to be the geometric effect of the tool and process geometry as well as the effect of the minimum chip thickness, which refers to the minimum thickness required for a chip to form. These are additional input parameters for the creation of the neural network for the prediction of the machining performance of AISI 630 steel. Vogler et al. (2005b) [8], in the second part of their paper, continued to use the minimum chip thickness to predict the cutting forces involved in the end milling operation. By assuming elastic deformation to occur when the chip thickness is less than the minimum chip thickness concept, they were able to achieve fairly accurate output values for the forces predicted. 

Peng et al. (2019) [9] conducted a combined study using 2-dimensional finite element analysis and comparing the obtained values to those obtained experimentally. The dataset was then used to train a neural network to obtain values for the specific cutting forces involved in the machining process. The authors convey that the developed network may not be able to be generalized as it is very sensitive to the hyperparameters involved; this led them to vary the depth and complexity of the neural network to increase the accuracy of the network as they continue to train the network on FEM data. This provides a comparable baseline for the methodology of this current research, as it is conducted in a very similar manner to the network proposed in this paper. Serin et al. (2017) [10] discussed the future of predictive analytics through the application of deep learning, and the shift towards Industry 4.0 by establishing metrics based on quality, performance, and energy consumption. They used variance analysis to determine the effect of input parameters on the desired output parameters, which are based on machining quality, performance, and energy consumption. They suggested that using training data from specific machining processes allows for the creation of highly accurate models. The use of this variance analysis enables us to determine which input parameters can be pruned for specific output variables when developing the general neural network for the machining of AISI 630 steel.

Shi et al. (2019) [11] established a new framework for the use of deep learning in tool condition monitoring through the use of parallel networks, and algorithms to simplify training data. The results of this new framework are shown to have good generalization capabilities compared to existing models being used, which implies that the use of multiple networks to associate inputs with a smaller group of output variables are likely to be more effective in terms of generalizing the use of the neural network. This method strongly emphasizes the need for a large quantity of quality training data, as with all deep learning approaches due to error propagation, etc. Gerum et al. (2020) [12] investigated the effect of sparsity in neural networks upon observing the model sparsity present in biological neural networks (i.e., the brain). Their findings suggested that the introduction of model sparsity during the training process improves the generalization ability of the neural network. The generalization of neural networks has been one of the larger issues faced in previous studies involving neural networks and metal cutting, as observed by Dimla Sr. and Lister (2000b) [4] and Peng et al. (2019) [9], so the ability to reduce the impact of this issue is definitely worth looking into.

Jian et al. (2018) [13] effectively used end to end deep learning to solve a previously difficult to solve problem in terms of the noise correction of infrared images. Through the use of multiple neural networks to specialize in solving the nonuniformity correction problem by simulating training data, the neural network is able to accurately estimate where the noise actually is. This can also be applied to the metal cutting problem, as the cutting parameters that greatly affect a large number of outputs, seen in the paper by Dimla Sr. and Lister (2000a) [3], can be applied as the inputs to multiple different neural networks to model their effect on these output variables separately in order to better simulate the more complex output conditions more quickly. Piotrowski and Napiorkowski (2013) [14] discussed the implementation of an Optimization Approximation Algorithm, which enables the neural network to identify when overfitting is occurring during training, which could heavily assist in proving the generalized neural network’s trustworthiness when solving for novel data.

Salimiasl and Ozdemir (2016) [15] compared the usage of artificial neural networks and fuzzy logic inference systems to predict tool flank wear via the use of cutting forces. They found that, when large datasets were available, artificial neural networks were the most accurate method of modeling real world data via the use of machine learning methods, specifically by studying the turning process via a full factorial design, following which the input and output data obtained by empirical–analytical modeling were used to train multiple styles of machine learning. With regards to Artificial Neural Networks, the network used had three layers, consisting of 21 neurons (16 neurons in the input layer, 4 neurons in the hidden layer, 1 neuron in the output layer). The number of neurons was determined via Kolmogorov’s theorem, as described in the paper, where two times the number of input nodes plus one is enough to calculate continuous functions. The authors found that Artificial Neural Networks returned the highest accuracy of results (R2 = 0.952), although there was a noticeable decrease in the accuracy when a small number of experiments was used to train the model.

Pai and Nagabhushana (2020) [16] conducted research into the applications of Artificial Neural Networks in Tool Condition Monitoring, and found that although Artificial Neural Networks (specifically Multi-Layer Perceptron) were very robust, compact, and able to predict tool wear with a high degree of accuracy, they still suffered from issues such as requiring a long training time and the issue of getting trapped in local minima, which could skew the model’s predictions. The experiment was designed around the milling process, with a focus on laying the groundwork for the development of a completely unmanned machining station involving real time monitoring, interpretation, and decision-making with regards to tool wear in order to avoid the additional costs incurred by tool breakage. With regards to the Artificial Neural Network used in this study, a three-layer neural network with a total of 37 neurons (12 24 1) was used, obtaining accuracy rates as high as 93 percent, though this accuracy rate was highly variable depending on the training data provided to the network and the disparity from the test data.

Mukherjee and Das (2021) [17] studied the use of Artificial Neural Networks for tool condition monitoring, specifically for use in an online monitoring system. They aimed to reduce the need for expensive sensor equipment by solely using the spindle speed as the primary wear detection parameter, although this would mean every other variable, such as the material being cut, etc., would have to be kept consistent, or a separate model would have to be built for every condition. However, in a mass-production context it may be assumed that all parameters will be kept mostly consistent, potentially lending viability to the system described in this paper. The study was conducted into the turning process, with the RPM being measured using infrared sensors, and tool wear being quantified in microns. The artificial neural network used was a network with two hidden layers, consisting of 54 neurons (4 32 16 2) using a dataset of 24 experiments. The authors found that although the neural network was able to make good estimates at low deviation, as the flank wear increased, so too did the neural network’s inaccuracy. They estimated this was due to the increase in contact area as the flank wear of the tool increased but concluded that this approach was still viable if cutting speed was kept to low variation and at a low deviation of tool wear. This minimal data approach would need to be tested further in a variety of situations, however, as there are several uncontrolled variables that may affect the tool wear in actual circumstances found in the industry.

Patra et al. (2017) [18] studied the potential use of Artificial Neural Networks for Tool Condition Monitoring, particularly for peck drilling in micro-machining. They describe how the condition of the tools used in micro-machining heavily affect the quality of the machined part, and therefore the necessity of being able to effectively monitor/predict the tool wear of the drills being used. By using the thrust force signals obtained via sensors as well as the tool wear measured via microscope for the neural network, experimental data via a full factorial experimental design using cutting speed and feed were obtained. These data were then fed into an Artificial Neural Network consisting of one hidden layer with 20 neurons (9 10 1) after being split into training, validation, and testing data (70-15-15 split), in order to ensure that overfitting of the network did not occur. The generated model from the Artificial Neural Network designed to output hole number from the average thrust force returned an R2 value of 0.995, and even after new cutting conditions were tested with the model, the mean prediction error percentage averaged to less than five percent which the authors considered acceptable due to the nonlinearity of the cutting forces involved in micromachining via the size effect. The usage of neural network is increasing in all disciplines of engineering, as reported by Rafiee and Mirjalily (2020) [19], Roshani et al. (2021) [20], and Pourbemany et al. (2021) [21].

With regards to the previous work performed in this field, the results obtained are clear in showing the potential of the applications of neural networks in this area, particularly with regards to the accuracy of using these networks to model machining output in terms of tool wear and performance. It is also clear, however, that there has not been enough research carried out into how these neural networks and systems based on these networks could be implemented into manufacturing facilities, particularly in terms of cost to benefit, as the advantages of implementing these systems would need to outweigh the cost in terms of time and other disposable resources in order to be considered a feasible recommendation for any facility.

The goal of this study is to suggest practical implementations for the research already undertaken in the field by proposing a novel approach towards the implementation of neural networks in manufacturing facilities. The study evolved from its initial premise as unlike most approaches that have been seen in this field with regards to neural networks, where the machining inputs are fed to the network to receive the predicted outputs, this study proposes a backwards training model where the known outputs are fed to the model for real-time monitoring of input conditions and for the simulation of optimal input parameters.

The innovation in this research stems from the fact that most studies already performed in this field investigated the prediction of output parameters from given input conditions or predicting dependent events from the machining process (for example, predicting tool wear from monitored machining output). We propose a direct analysis of the machining performance based on the known material properties and known parameters of machining tools by feeding the network output data in order to ensure input conditions remain as expected during machining, or to establish the optimal input conditions prior to machining.

## 2. Hybrid Finite Element (FE)-Machine Learning (ML) Methodology

**Figure 1:**
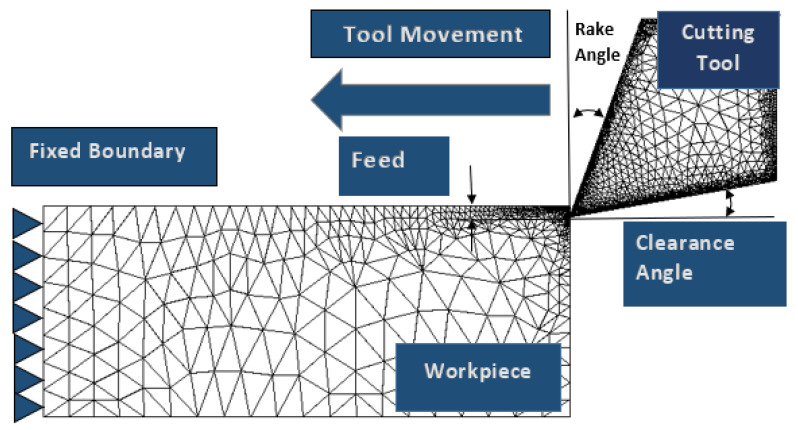
Schematic illustration of FE model setup.

**Figure 2:**
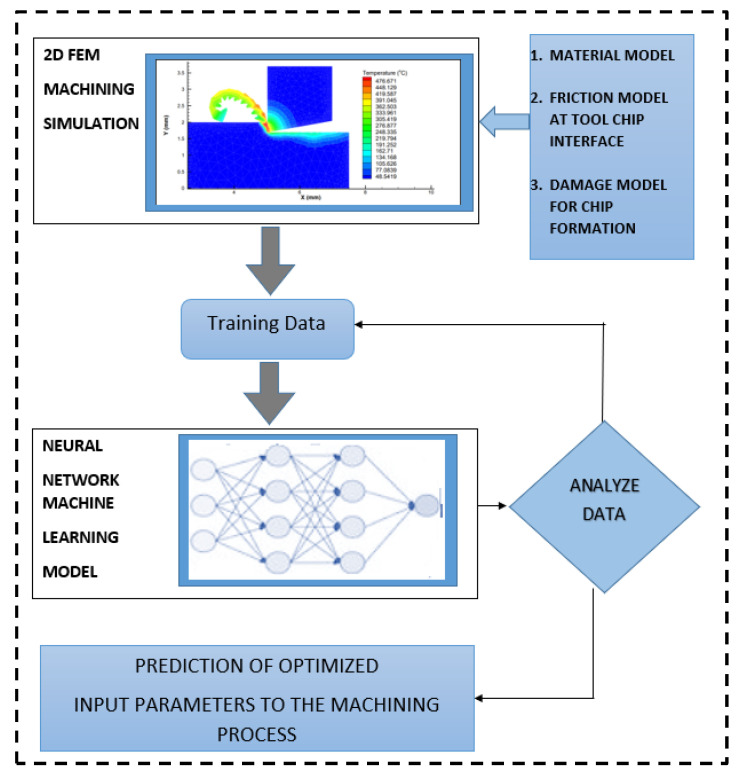
Flow diagram of adopted methodology.

The presented 2D Finite element numerical model was developed using AdvantEdge (7.13, Minneapolis and United States of America), a finite element solver developed by Third Wave Systems (TWS), Minneapolis, United States of America. Using AdvantEdge, we are able to simulate the 2D cutting process with chip formation. The solver works on explicit Lagrangian-assisted code to run coupled thermomechanical transient analysis, with chip formation as the result of remeshing and adaptive meshing. Figure 1 shows the schematic illustration of the model used in the AdvantEdge. The setup involved cutting tool as a carbide, an edge radius of 0.040 mm, and a rake angle of 0° in all simulations. Meshing of workpiece materials was based on the total number of 24,000 elements, maximum element size 0.1 and minimum element size of 0.02. To control distortions, mesh refinement factor and coarsening factors were used as two and six, respectively. Figure 2 shows the adopted methodology.

The software has several options to allow for the modeling of the selected workpiece material for this study (AISI630); however, in this study the material was modeled using an advanced representation of the power law. This representation has the ability to incorporate the damage law along with temperature-dependent thermal conductivity and heat capacity in the calculations for the workpiece material, as can be seen in the documentation by ThirdWaveSystems (2017) [22]. 

The adaptive remeshing triggers automatically when the elemental distortions exceed the specified tolerance. This meshing was continuously monitored, and the elemental distortion was fixed by further refinement and improvement as per Man et al. (2012) [23]. The mesh refinement factor and coarsening factors were set as two and six, respectively. The advanced power law used in this work is represented in Equation (1) as documented by ThirdWaveSystems (2017) [22]. The power law incorporates the strain hardening function as g($\varepsilon^{p}$), the thermal softening function as θ(T) and the rate sensitivity function is given by τ($\dot{\varepsilon }$).

(1) $$\sigma \;(\varepsilon^{p},T,\dot{\varepsilon })=g(\varepsilon^{p})\;\theta (T)\;\tau \dot{\varepsilon }$$

The modeling of the strain hardening function is represented by Equation (2). In order to utilize the function of strain hardening, the stress strain data of the workpiece material can be examined and fit to a curve. The strain rate sensitivity function is then modeled by Equation (3) as documented by ThirdWaveSystems (2017) [22]. To form precise chip shapes and cutting force behavior it is important that the model is also able to account for thermal softening behavior. The thermal softening function was introduced into the power law via the fifth order polynomial equation described in Equation (4) as documented by ThirdWaveSystems (2017) [22].

(2) $$g(\varepsilon^{p})=\sigma o\;{\left[1+\frac{\varepsilon^{p}}{\varepsilon^{p}o}\right]}^{\frac{1}{n}}\;if\;\varepsilon^{p}<\varepsilon_{cut}^{p}$$

In Equation (2), *σo* is the initial yield stress, *p* is plastic strain, $\epsilon_{o}^{p}$ is the reference plastic strain, $\frac{1}{n}$ is the strain hardening power. The values of initial yield stress can be obtained from the stress strain data from the uniaxial compression or tensile tests of the workpiece material. The values of $\epsilon_{o}^{p}$ and n is obtained by curve fitting the stress strain data.

The AdvantEdge software manual and literature by Laakso and Niemi (2016) [24] and Ebrahimi et al. (2019) [25] were consulted to obtain all the required parameters for the AISI 630 material model. The strain hardening component of the workpiece material model was modeled by the fitting of a stress–strain curve for AISI 630.

(3) $$\tau (\dot{\varepsilon })=\sigma o{\left[1+\frac{\dot{\varepsilon }}{\dot{\varepsilon }o}\right]}^{\frac{1}{m1}}$$

The function in Equation (3) represents the flow stress behavior at higher strain rates. In this equation ε is the plastic strain rate, and $\dot{\varepsilon }o$ is the reference plastic strain rate, with *m*1 as the strain rate sensitivity.

*θ*(*T*) = *c*_0_ + *c*_1_*T*^1^ + *c*_2_*T*^2^ + *c*_3_*T*^3^ + *c*_4_*T*^4^ + *c*_5_*T*^5^  *if T* < *T_cut_* (4)

(5) $$\theta (T)=\;\theta (T_{\mathrm{cut}})\;(1-\frac{T-T_{cut}}{T_{m}-T_{cut}})\;if\;T>T_{cut}$$

In Equations (4) and Equation (5), the thermal softening function has been represented. The thermal softening function is composed of a fifth order polynomial with five constants denoted by *c*. These constants are curve fitted by the compression test data at elevated temperatures, where *T* is the temperature during the test, *T_m_* is the melting temperature, and *T_cut_* is the cut-off temperature. AdvantEdge uses the classical sliding friction concept to model the friction at the tool chip contact. The theory is based on the directly proportional relationship between frictional sliding force and the normal load. The ratio of these forces is taken as the coefficient of friction µ and taken as a constant value along the tool chip contact length as in Equation (6). The value of friction coefficient was implemented through Equation (7) as seen in Maranhão and Paulo Davim (2010) [26].

*T* = *μ σ_n_* (6)

(7) $$\mu =\frac{F_{c}\;tan\alpha +F_{t}}{F_{c}-F_{t}tan\alpha }$$

**Figure 3:**
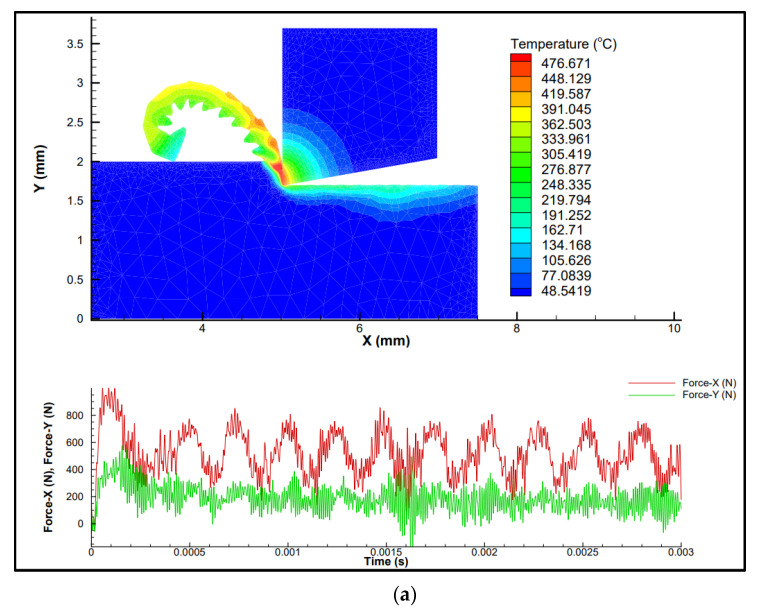

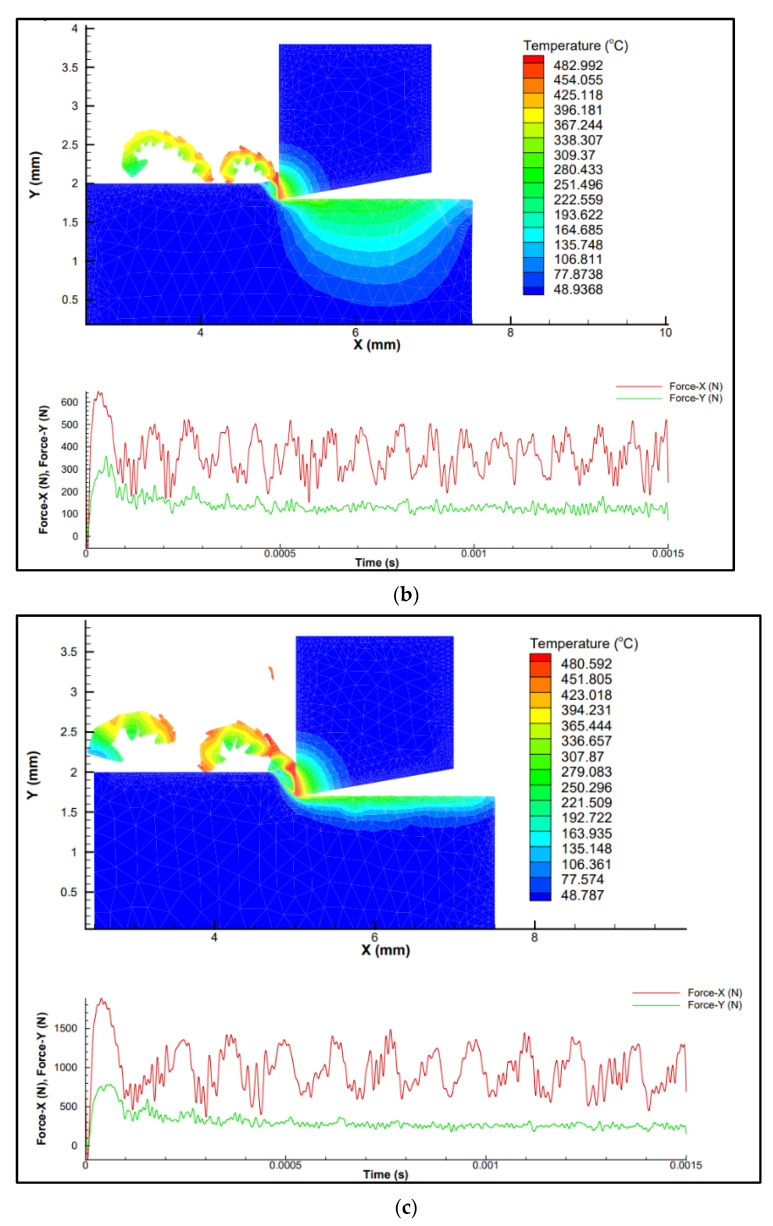

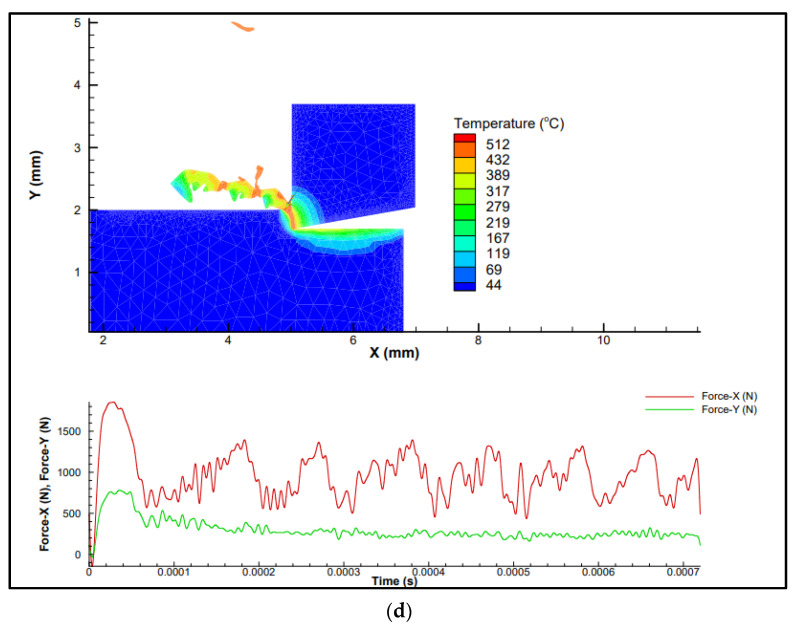
Finite element simulation (**a**) v = 50 mm/min, feed = 0.3 mm/rev, DoC = 1 mm, (**b**) v = 100 mm/min, feed = 0.2 mm/rev, DoC = 1 mm, (**c**) v = 100 mm/min, feed = 0.3 mm/rev, DoC = 2 mm, (**d**) v = 150 mm/min, feed = 0.3 mm/rev, DoC = 2 mm.

Another key parameter with regards to the chip shape is the damage in the workpiece material. AdvantEdge is able to simulate damage in the workpiece material by using a damage function D. The damage function D is shown in Equation (8). The fracture strain can be represented using a temperature-dependent model as shown in Equation (9), as documented by ThirdWaveSystems (2017) [22]. Table 1 shows FEM inputs. A full factorial design using a combination of these inputs was modeled for 27 sets of time series data. Figure 3 shows some sample simulations from the design of experiments.

(8) $$D=\underset{i}{\sum }\frac{\Delta \varepsilon_{i}^{p}}{\varepsilon_{f_{i}}^{p}}$$

(9) $$\varepsilon_{fo}^{p}=d_{o}+d_{1}T^{1}+d_{2}T^{2}+d_{3}T^{3}+d_{4}T^{4}+d_{5}T^{5}$$

AdvantEdge was used to run 27 simulations as seen in Figure 2 in a full factorial design with three variations of the cutting speed vs., feed rate f, and depth of cut d, to obtain the outputs of force in the x direction Fx, force in the y direction Fy, power P, and temperature T. The complete design of experiments used can be found in Table 2. These 27 sets of time series data were then processed as described in the following section.

Table 2: Design of experiments involved. The whole time series output data were fed to the network; however, an average of these outputs have been presented here for visualization purposes.

The machine learning model used in this study utilizes a Multi-Layer Perceptron (MLP) classifier, which means that the input signals are processed through a number of hidden layers consisting of a number of nodes through which they are processed, providing the requested output values. This model optimizes the log-loss function by stochastic gradient descent, specifically an optimizer named “adam” which according to Pedregosa et al. (2011) [27] was proposed by Kingma, Diederik, and Ba. The activation function used in this model is the rectified linear unit function as detailed by Pedregosa et al. (2011) [27], which can be found in Equation (10).

*f*(*x*) = *max* (0, *x*) (10)

**Figure 4:**
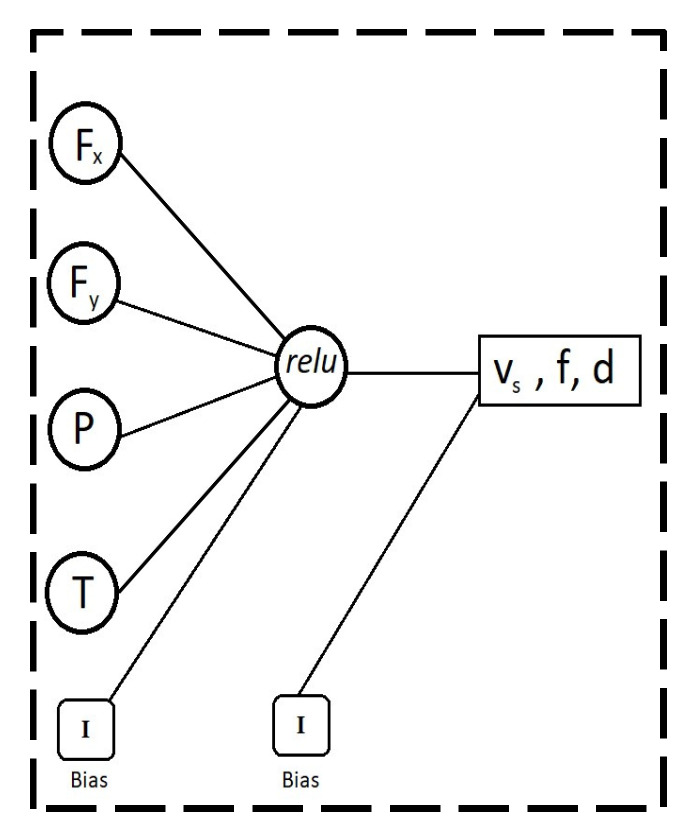
Representation of neural network.

The MLP classifier used in this study consisted of a single hidden layer with a single node to reduce computational time and power requirements. The representation in Figure 4 provides an overview of the model. The training data obtained through the finite element modeling were then split at a ratio of approximately 2:1 training data to validation data (with 7 sets as validation data and 20 sets as training data). The resultant network was trained and tested and the predicted outputs were then compared to the actual outputs and the accuracy score was calculated based on the percentage of values that were accurately predicted. Due to the size of the dataset extracted from the simulated environment, cross-validation techniques and dimensionality reduction techniques were applied. This ensured that the model developed was not biased and over-fitting to the test and training data.

In this study, the input data for the machine learning model were taken as the simulated outputs of force in the x direction Fx, force in the y direction Fy, power (P), and temperature (T). By placing the outputs as the cutting speed (vs.), feed rate (f), and depth of cut (d), this provides us the ability to feed this trained model the maximum tolerances for the forces being applied, power, and temperature and get the optimal values for vs., f, and d. This backwards model was used in order to take advantage of a neural network’s ability to process complex data into simple outputs. This model also allows for the desired final output to be given to the trained model in order to receive the ideal input parameters for the desired output, which is often the case in manufacturing, where the desired output is known, but the inputs needed to achieve said outputs requires specialized expertise in working with the material of manufacture.

## 3. Results and Discussion

**Figure 5:**
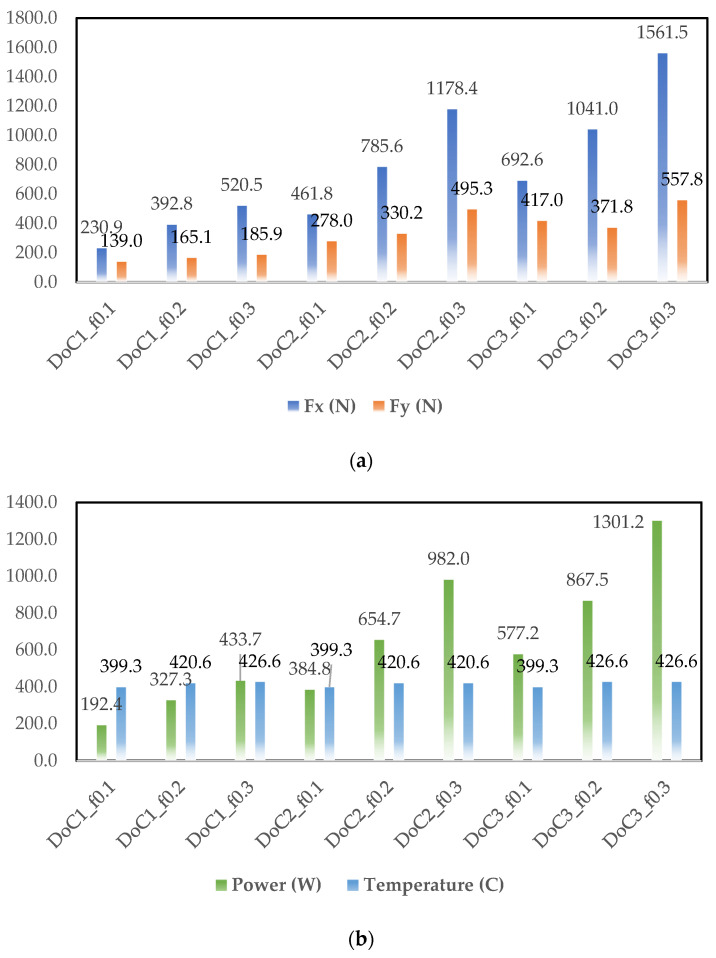
Simulated output parameters obtained for the cutting speed of 50 m/min (**a**) Fx and Fy cutting forces, (**b**) power (W), and temperature (°C).

**Figure 6:**
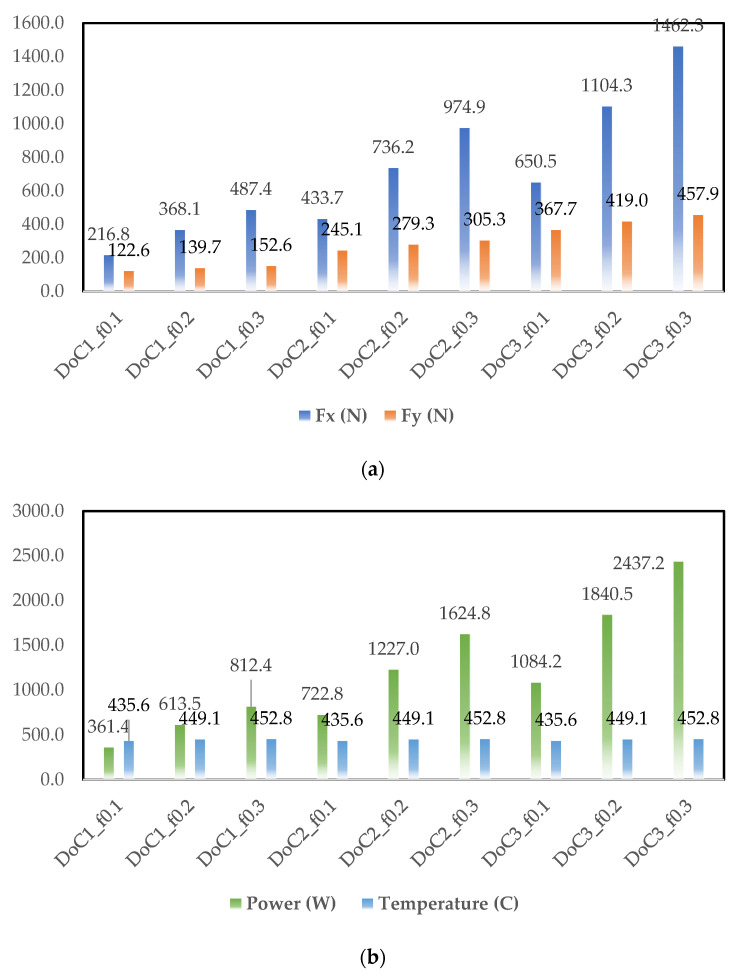
Simulated output parameters obtained for the cutting speed of 100 m/min (**a**) Fx and Fy cutting forces, (**b**) power (W), and temperature (°C).

**Figure 7:**
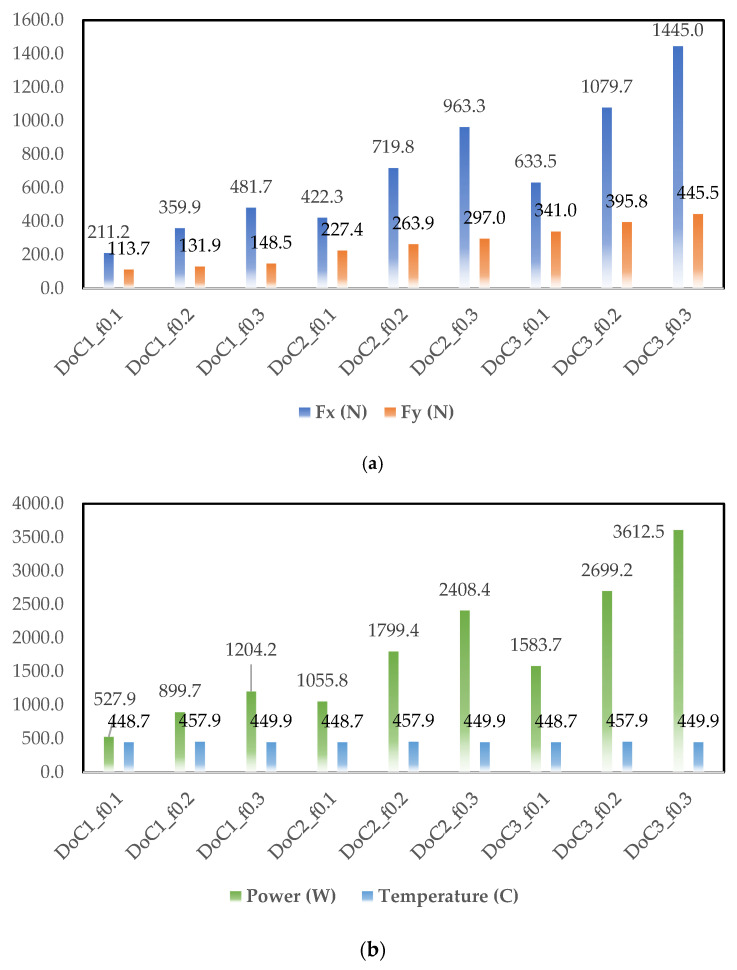
Simulated output parameters obtained for the cutting speed of 150 m/min (**a**) Fx and Fy cutting forces, (**b**) power (W), and temperature (°C).

Based on the simulation results, cutting force components (Fx and Fy), power, and cutting temperature are plotted in Figure 5, Figure 6 and Figure 7. As observed in Figure 3, Figure 4, Figure 5 and Figure 6, at different cutting speeds ranging from 50–150 m/min it can be seen that cutting forces and power increased with the increase in the feed rate and depth of cut (DoC). However, cutting temperature fluctuation was in the range of 30–40 °C.

The increase in the cutting forces and power with increasing feed level is linked with higher frictional contact and it is found in agreement with the traditional metal cutting literature. The other trend of cutting forces and power was related to the depth of cut. Higher depth of cut provided higher forces and power due to the presence of higher chip load. Increasing the cutting speed from 50–150 m/rev lowered the cutting force component and the behavior can be linked with the thermal softening behavior of the material. This behavior was also found in agreement with the metal cutting literature because a higher cutting speed increases temperature at the tool chip interface.

Once the model was trained and tested, the accuracy scores and time taken for training and results obtained were as seen in Table 3. The results reflected a high degree of accuracy when predicting the cutting speed, although this accuracy took a sharp dip when predicting feed rate f, although the depth of cut d prediction returned a fairly high accuracy level. This was due to the large variation in the cutting speed, making it easier on the neural network to detect the changes occurring due to the difference in cutting speed. On this note, if the study were to be repeated, then the difference in the feed rate and depth of cut would have to be increased to effectively train the neural network.

It is likely that the change in the cutting speed more strongly affected the forces applied, power, and temperature compared to the variation in the feed rate and depth of cut. The model would have to be trained further with a greater volume of data in order to be used effectively for predicting depth of cut and feed rate. Another key point to note is that although each finite element simulation took approximately 2 h, the single hidden layer neural networks were able to complete training and predict the output values within a minute each.

**Figure 8:**
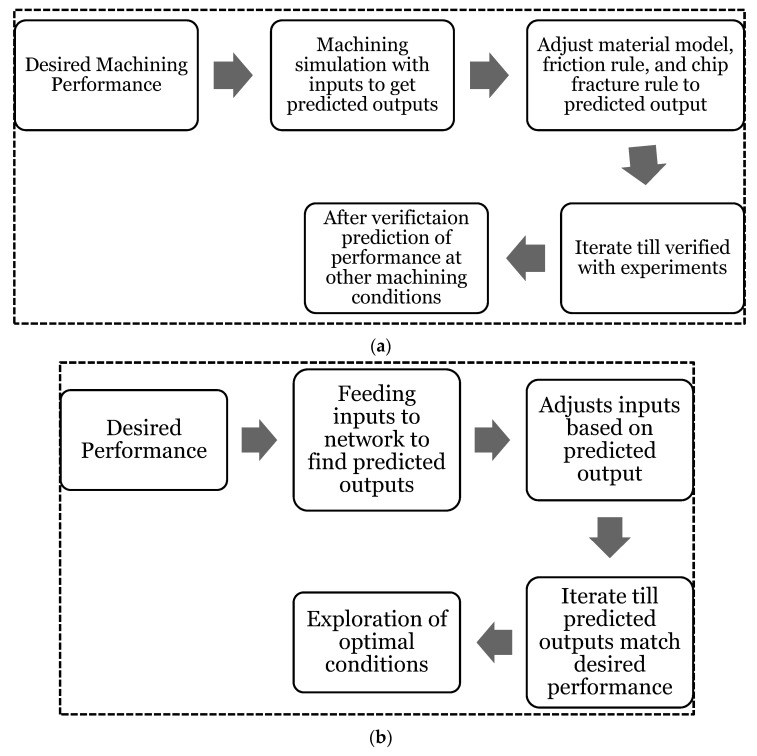

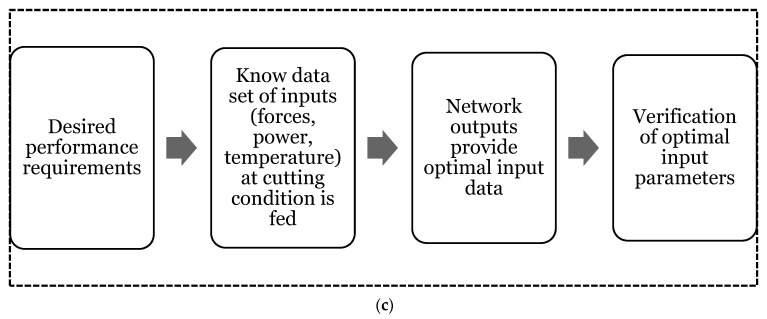
(**a**) Workflow of finite element simulation; (**b**) W = workflow with MLP-Neural Network; (**c**) proposed workflow with backward neural network.

Figure 8a–c provide the workflow of finite element simulation, a conventional neural network and the proposed backward neural network. This backwards trained model would be ideal in the case real time monitoring output data were available through the use of dynamometers and other sensors. For clarity, we can consider the workflow of the standard approach to machining data and MLP Networks as well as why they would be used: the manufacturer desires certain output conditions (the dependent variables), and has control over the input conditions, which (as simplified here) are the cutting speed, feed rate, and depth of cut (the independent variables). The manufacturer feeds various input conditions to the network in order to see the predicted output conditions and whether they fit within the allowable tolerances. The network can provide the output condition much faster than a simulation would be able to.

If the backwards approach could be proven viable through experimentation, it is also possible that manufacturers may be able to input their maximum tolerances for forces applied, power generated, and temperature of the workpiece for AISI 630 steel, and obtain the optimum initial parameters for machining in a very short amount of time as opposed to waiting hours for a simulation to run. This could also hold implications for real-time monitoring systems for machining AISI 630 steel.

## 4. Conclusions

In conclusion, although applied to a limited dataset, it is the observation of the authors that this method of determining optimal parameters for machining shows great promise. The results obtained show that there is scope for accurately predicting input conditions based on the machining output, especially with regards to the cutting speed and depth of cut, which brings us to the limitations of this concept.

Although, as expected, cutting forces and power increased by increasing the feed rate and depth of cut, but lowered by increasing the cutting speed due to higher friction, higher chip load, and thermal softening, respectively, the effect of the cutting speed was far more significant on the outputs than the other two input conditions. This large effect of the cutting speed on the output conditions does mean that the model is less reliable when predicting the other input conditions, however this may not be the case if a greater dataset is used with more significant variation in the other input parameters.

Another limitation of this system is that it is only able to accurately predict data within the limits of the training data provided to it; for example, if the training data used only have examples of cutting speeds from 50 m/min to 150 m/min, then any predictions obtained when the network is fed data at 200 m/min will likely be wildly inaccurate as the network itself lacks a frame of reference for these out of scope values. The effect of this, however, is mitigated by the fact that all training data will be limited to the capacity of the machine they are trained for. After all, if the tool itself is unable to reach out of scope values, then it is impossible for the network to ever be fed out of scope values.

In practical implementation, manufacturers of AISI 630 steel would be able to input their previous machining output data, paired with their input conditions (assuming the availability of previous sensor data), such as levels for forces applied, power generated, and temperature of the workpiece, in order to train the model. The trained model would then be able to provide the optimal input parameters that should be used based on the manufacturer’s desired output conditions, such as limitations in power supplied, desire to keep shear forces to a minimum, desire to limit temperature rise, etc. This method of using the network would cut hours of the time required to run FEA simulations to ensure that output conditions do not exceed the known limits of the facility. As the network itself is also less computationally intensive than running FEA simulations, it would also allow for smaller facilities to run “simulations” at a similar pace to larger facilities.

The results obtained also show promise for Industry 4.0, with online real time monitoring in order to detect anomalies through the application of 5G technology and narrow-band Internet of Things. By feeding real time sensor output data to the network, the expected input conditions can be monitored and large variations from what is expected can be quickly detected and the system shut down to prevent injury, harm, or the destruction of the workpiece.

In order to further establish the viability of the proposed model, however, it is critical that the methodology is applied to real world experimental data, as the ability to predict experimental data based on the network trained on simulation data will be the true test of this method’s feasibility. It is also necessary to look further into which input conditions would be best suited to be predicted with this model, as the viability of predicting the feed rate from this model has been shown to be very low. Alternatively, further study could be performed into which output conditions would be best to predict particular inputs, therefore potentially leading to multiple networks working in tandem to monitor individual input conditions.

It is clear, in the end, that neuraTablel networks and machine learning are powerful tools in the engineer’s arsenal, but must be used creatively to obtain data that can be implemented for industrial applications.

## Figures and Tables

**Table 1:** Finite element modeling inputs.

Cutting Speed (*v_s._*) (m/min)	Feed Rate (*f*) [mm/rev]	Depth of Cut (*d*) [mm]
50	0.1	1
100	0.2	2
150	0.3	3

**Table 2:** Design of experiments with input and response parameters.

Sr. No.	Cutting Speed (m/min)	Feed (mm/rev)	Depth of Cut (mm)	Fx (N)	Fy (N)	Power (W)	Temperature (°C)
Sim1	50	0.1	1	230.9	139.0	192.4	399.3
Sim2	50	0.1	2	461.8	278.0	384.8	399.3
Sim3	50	0.1	3	692.6	417.0	577.2	399.3
Sim4	50	0.2	1	392.8	165.1	327.3	420.6
Sim5	50	0.2	2	785.6	330.2	654.7	420.6
Sim6	50	0.2	3	1178.4	495.3	982.0	420.6
Sim7	50	0.3	1	520.5	185.9	433.7	426.6
Sim8	50	0.3	2	1041.0	371.8	867.5	426.6
Sim9	50	0.3	3	1561.5	557.8	1301.2	426.6
Sim10	100	0.1	1	216.8	122.6	361.4	435.6
Sim11	100	0.1	2	433.7	245.1	722.8	435.6
Sim12	100	0.1	3	650.5	367.7	1084.2	435.6
Sim13	100	0.2	1	368.1	139.7	613.5	449.1
Sim14	100	0.2	2	736.2	279.3	1227.0	449.1
Sim15	100	0.2	3	1104.3	419.0	1840.5	449.1
Sim16	100	0.3	1	487.4	152.6	812.4	452.8
Sim17	100	0.3	2	974.9	305.3	1624.8	452.8
Sim18	100	0.3	3	1462.3	457.9	2437.2	452.8
Sim19	150	0.1	1	211.2	113.7	527.9	448.7
Sim20	150	0.1	2	422.3	227.4	1055.8	448.7
Sim21	150	0.1	3	633.5	341.0	1583.7	448.7
Sim22	150	0.2	1	359.9	131.9	899.7	457.9
Sim23	150	0.2	2	719.8	263.9	1799.4	457.9
Sim24	150	0.2	3	1079.7	395.8	2699.2	457.9
Sim25	150	0.3	1	481.7	148.5	1204.2	449.9
Sim26	150	0.3	2	963.3	297.0	2408.4	449.9
Sim27	150	0.3	3	1445.0	445.5	3612.5	449.9

**Table 3:** Computational aspects of results of proposed modeling.

Output	Accuracy [%]	Time Taken [s]
Vs.	99	39
F	19	62
DoC	86	64

## Data Availability

The data that support the findings of this study are available on request from the corresponding author, [S.P.].

## References

[1] Serin G., Sener B., Ozbayoglu A.M., Unver H.O. (2020). Review of tool condition monitoring in machining and opportunities for deep learning. Int. J. Adv. Manuf. Technol..

[2] Bianchini M., Scarselli F. (2014). On the Complexity of Neural Network Classifiers: A Comparison Between Shallow and Deep Architectures. IEEE Trans. Neural Netw. Learn. Syst..

[3] Dimla D.E., Lister P. (2000). On-line metal cutting tool condition monitoring.: I: Force and vibration analyses. Int. J. Mach. Tools Manuf..

[4] Dimla D.E., Lister P. (2000). On-line metal cutting tool condition monitoring.: II: Tool-state classification using multi-layer perceptron neural networks. Int. J. Mach. Tools Manuf..

[5] Kumar M., Datta S., Kumar R. (2018). Electro-discharge Machining Performance of Ti–6Al–4V Alloy: Studies on Parametric Effect and Phenomenon of Electrode Wear. Arab. J. Sci. Eng..

[6] Samy G., Kumaran S., Uthayakumar M. (2021). Observations of machining performance during turning AA6351-B4C composite. J. Test. Eval..

[7] Vogler M.P., Devor R.E., Kapoor S.G. (2004). On the Modeling and Analysis of Machining Performance in Micro-Endmilling, Part I: Surface Generation. J. Manuf. Sci. Eng..

[8] Vogler M.P., Kapoor S.G., Devor R.E. (2004). On the Modeling and Analysis of Machining Performance in Micro-Endmilling, Part II: Cutting Force Prediction. J. Manuf. Sci. Eng..

[9] Peng B., Bergs T., Schraknepper D., Klocke F., Döbbeler B. (2019). A hybrid approach using machine learning to predict the cutting forces under consideration of the tool wear. Procedia CIRP.

[10] Serin G., Gudelek M.U., Ozbayoglu A.M., Unver H.O. Estimation of parameters for the free-form machining with deep neural network. Proceedings of the IEEE International Conference on Big Data (Big Data).

[11] Shi C., Panoutsos G., Luo B., Liu H., Li B., Lin X. (2019). Using multiple-feature spaces-based deep learning for tool condition monitoring in ultraprecision manufacturing. IEEE Trans. Ind. Electron..

[12] Gerum R.C., Erpenbeck A., Krauss P., Schilling A. (2020). Sparsity through evolutionary pruning prevents neuronal networks from overfitting. Neural Netw..

[13] Jian X., Lv C., Wang R. (2018). Nonuniformity Correction of Single Infrared Images Based on Deep Filter Neural Network. Symmetry.

[14] Piotrowski A.P., Napiorkowski J. (2013). A comparison of methods to avoid overfitting in neural networks training in the case of catchment runoff modelling. J. Hydrol..

[15] Salimiasl A., Özdemir A. (2016). Analyzing the performance of artificial neural network (ANN)-, fuzzy logic (FL)-, and least square (LS)-based models for online tool condition monitoring. Int. J. Adv. Manuf. Technol..

[16] Pai S.P., Nagabhushana N.T. (2020). Handbook of Research on Emerging Trends and Applications of Machine Learning. Tool Condition Monitoring Using Artificial Neural Network Models.

[17] Mukherjee A., Das S. (2021). A simple online tool condition monitoring system using artificial neural networks. IOP Conf. Series: Mater. Sci. Eng..

[18] Patra K., Jha A., Szalay T., Ranjan J., Monostori L. (2017). Artificial neural network based tool condition monitoring in micro mechanical peck drilling using thrust force signals. Precis. Eng..

[19] Rafiee P., Mirjalily G. (2020). Distributed Network Coding-Aware Routing Protocol Incorporating Fuzzy-Logic-Based Forwarders in Wireless Ad hoc Networks. J. Netw. Syst. Manag..

[20] Roshani M., Phan G., Faraj R.H., Phan N.-H., Roshani G.H., Nazemi B., Corniani E., Nazemi E. (2021). Proposing a gamma radiation based intelligent system for simultaneous analyzing and detecting type and amount of petroleum by-products. Nucl. Eng. Technol..

[21] Pourbemany J., Essa A., Zhu Y. (2021). Real Time Video based Heart and Respiration Rate Monitoring. arXiv.

[22] ThirdWaveSystems (2017). Third Wave AdvantEdge TM User’s Manual Version 7.3.

[23] Man X., Ren D., Usui S., Johnson C., Marusich T.D. (2012). Validation of Finite Element Cutting Force Prediction for End Milling. Procedia CIRP.

[24] Laakso S.V.A., Niemi E. (2016). Modified Johnson–Cook flow stress model with thermal softening damping for finite element modeling of cutting. Proc. Inst. Mech. Eng. Part B J. Eng. Manuf..

[25] Ebrahimi S.M., Araee A., Hadad M. (2019). Investigation of the effects of constitutive law on numerical analysis of turning processes to predict the chip morphology, tool temperature, and cutting force. Int. J. Adv. Manuf. Technol..

[26] Maranhão C., Paulo Davim J. (2010). Finite element modelling of machining of aisi 316 steel: Numerical simulation and ex-perimental validation. Simul. Model Pract. Theory.

[27] Pedregosa F., Varoquaux G., Gramfort A., Michel V., Thirion B., Grisel O., Blondel M., Prettenhofer P., Weiss R., Dubourg V. (2011). Scikit-learn: Machine learning in Python. J. Mach. Learn. Res..

